# Global perspectives on the biodegradation of LDPE in agricultural systems

**DOI:** 10.3389/fmicb.2024.1510817

**Published:** 2025-01-07

**Authors:** Jani E. Mendoza, Daniel Tineo, Beimer Chuquibala-Checan, Nilton Atalaya-Marin, Victor H. Taboada-Mitma, Josué Tafur-Culqui, Ever Tarrillo, Darwin Gómez-Fernández, Malluri Goñas, María Andrea Reyes-Reyes

**Affiliations:** ^1^Centro Experimental Yanayacu, Dirección de Supervisión y Monitoreo en las Estaciones Experimentales Agrarias (DSME), Instituto Nacional de Innovación Agraria (INIA), Lima, Peru; ^2^Corporación para la Investigación de la Corrosión (CIC), Piedecuesta, Colombia; ^3^Grupo de Investigación en Compuestos Orgánicos de Interés Medicinal (CODEIM), Universidad Industrial de Santander (UIS), Bucaramanga, Colombia

**Keywords:** microbial consortia, plastic degradation pathways, HPLC, enzymatic biodegradation, metagenomic, SEM, FTIR, biological waste management

## Abstract

The increasing use of plastics globally has generated serious environmental and human health problems, particularly in the agricultural sector where low-density polyethylene (LDPE) and other plastics are widely used. Due to its low recycling rate and slow degradation process, LDPE is a major source of pollution. This paper addresses the problem of plastic accumulation in agriculture, focusing on LDPE biodegradation strategies. The studies reviewed include recent data and the methodologies used include state-of-the-art technologies and others that have been used for decades, to monitor and measure the degree of biodegradation that each treatment applied can have, including SEM, GCMS, HPLC, and microscopy. The countries investigating these biodegradation methodologies are identified, and while some countries have been developing them for some years, others have only begun to address this problem in recent years. The use of microorganisms such as bacteria, fungi, algae, and insect larvae that influence its decomposition is highlighted. A workflow is proposed to carry out this type of research. Despite the advances, challenges remain, such as optimizing environmental conditions to accelerate the process and the need for further research that delves into microbial interactions in various environmental contexts.

## 1 Introduction

Plastic production worldwide has increased since 1950, the time when large-scale production started, reaching more than 368 million tons reported in 2019 and 2020 (Ekanayaka et al., 2022; Plastics-Europe, 2021; Temporiti et al., 2022). Global consumption of plastics will continue to increase, so much so that by 2050 it is estimated that there will be up to 5.3 gigatons of plastics discarded in the environment (Schwarz et al., 2023). More than 90% of discarded plastic is not pretreated and goes into the sea and landfills (UNDP, 2023). This accumulation is evidenced by macro and microplastics, representing an environmental and public health problem, with high accumulation capacity in different ecosystems. This affects agricultural and livestock development and human health, causing various pathologies and in some cases even the death of different living beings (Hale et al., 2020; Skariyachan et al., 2021; Yee et al., 2021; Welden, 2020). Most of these materials are non-biodegradable, highly efficient, and versatile, exhibiting a range of applications, due to their distinctive physical, chemical, mechanical, and thermal properties with high resistance to atmospheric and corrosive agents, obtained from petroleum derivatives and other hydrocarbons (Ali et al., 2018). The most widely applied and therefore the main plastic wastes are low-density polyethylene (LDPE), high-density polyethylene (HDPE), polyethylene terephthalate (PET), polypropylene (PP), polyvinyl chloride (PVC), and polystyrene (PS) (Temporiti et al., 2022; Lear et al., 2021; Matjašič et al., 2021; Zeghal et al., 2021). After their use, plastics become solid waste, which depending on the treatment in each country can follow three processes: recycling, incineration as a source of energy, or accumulation in landfills (Temporiti et al., 2022; Lear et al., 2021; Buchholz et al., 2022). When accumulated, these wastes require long periods for their natural degradation. The condition associated with the inherent characteristics of the plastic, such as high molecular weight, crystallinity, hydrophobicity, and the additives used to improve the quality of the final product, favoring resistance to atmospheric and corrosive agents (Temporiti et al., 2022; Lear et al., 2021; Matjašič et al., 2021; Harshvardhan and Jha, 2013).

Modern agriculture in some countries of the world has become dependent on single-use and extended-use plastic products for crop production. These materials have facilitated increased productivity and efficiency in agriculture (Lakhiar et al., 2024). The most frequent uses of plastics are oriented to soil covering, greenhouses, irrigation systems, seed germination, production, and packaging of products (Hofmann et al., 2023). The massive use of these polymers has been generating several environmental problems because they take hundreds of years to degrade; this problem is aggravated by the low recycling rate of agricultural plastics, partly due to contamination with soil, crop residues, and agricultural chemicals (Lakhiar et al., 2024; Hofmann et al., 2023; Correa-Cano et al., 2023). The proper management of these wastes is related to Sustainable Development Goals 3, 12, and 15 (FAO, 2023).

**Figure 1:**
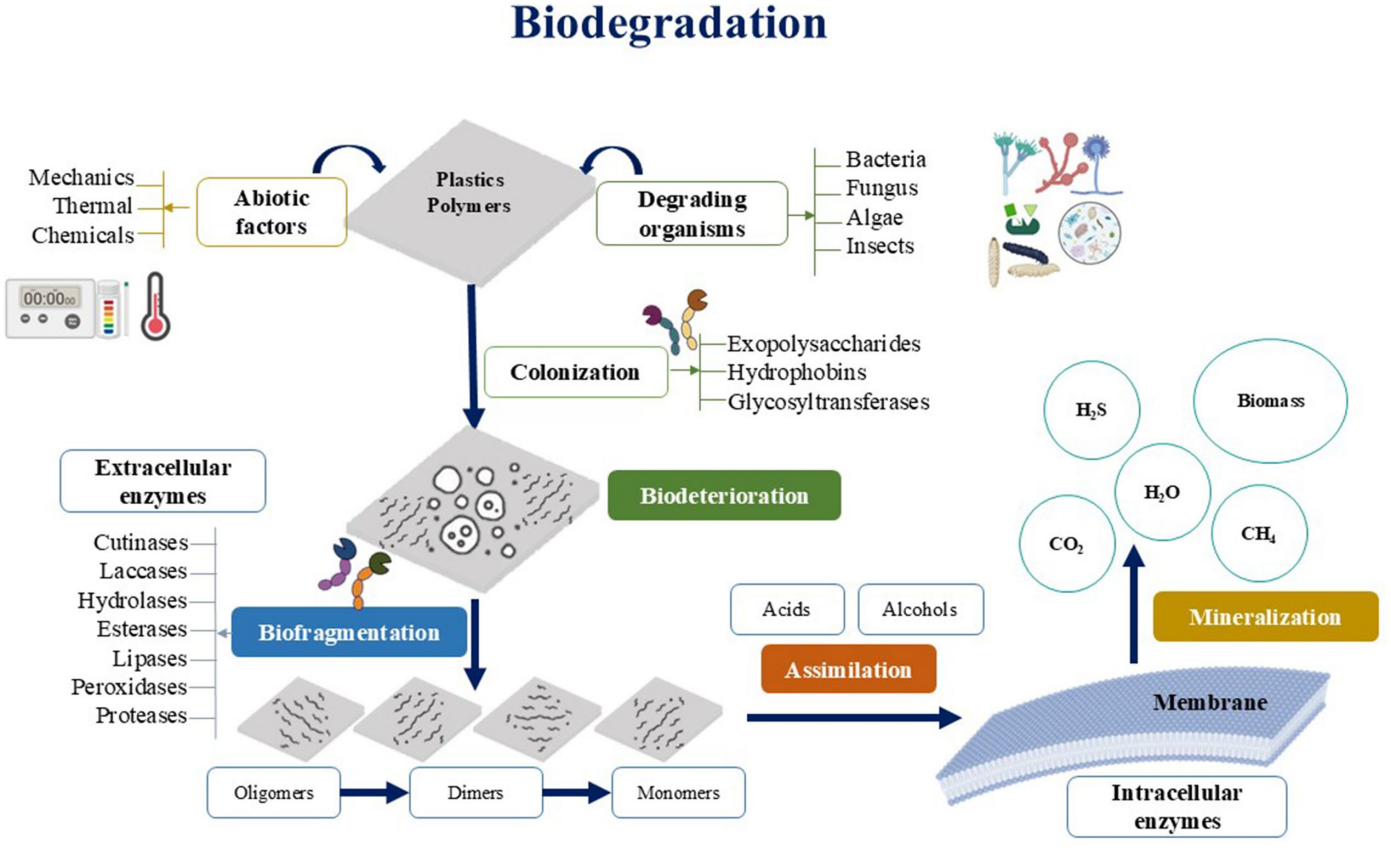
Processes in biodegradation.

In the search for green alternatives for managing plastic waste, biodegradation mechanisms are highlighted as a possible solution to mitigate this problem, particularly in the agricultural context. This biodegradation mechanism is a physicochemical and biological process mediated by microorganisms, such as bacteria, fungi, algae, and other organisms or substances of biological origin (Koh and Khor, et al., 2022; Jacquin et al., 2019; Rogers et al., 2020). The biodegradation mecanism has four main stages (see Figure 1): biodeterioration (colonization), biofragmentation, assimilation, and mineralization (Lucas et al., 2008), each influenced by a combination of environmental and microbial factors. In the biodeterioration phase, abiotic conditions such as temperature, humidity, UV radiation, and mechanical forces weaken the polymer matrix, creating favorable sites for microbial colonization (Nag et al., 2021; Islami et al., 2019; Chaudhary et al., 2021).

Since plastisphere, as plastics have come to be called on the planet, linger in nature after disposal, causing damage with consequences for wildlife and human life (Žuna Pfeiffer et al., 2022). Furthermore, being a developing research topic, methodologies for biodegrading and measuring the biodegradation of these plastics are still under investigation. This review article examines various organisms and methods employed in the biodegradation of plastics, with a particular focus on LDPE due to its widespread use in agriculture. Differences and similarities between methods are discussed, elucidating procedures that allow biodegradation to be measured accurately and reproducibly. In addition, the review discusses the challenges and opportunities associated with the implementation of these technologies in agriculture and reviews the organisms used for this purpose. The analysis presented in this manuscript can contribute to the development of a more sustainable and resilient agriculture in the face of global environmental challenges.

The term “plastisphere,” coined to describe the accumulation of plastics in the environment, has gained prominence in recent years. These persistent pollutants, often referred to as “plastics” on Earth, have been linked to adverse effects on wildlife and human health (Žuna Pfeiffer et al., 2022). Moreover, as this is a developing research area, these plastics’ biodegradation and measurement methodologies are still under investigation. This is the pioneering study to explore this topic. For Peru that examines the various organisms and methods utilized in the biodegradation of plastics, with a particular focus on LDPE due to its pervasive usage in agricultural applications, and discusses the differences and similarities between methods and procedures to accurately and reproducibly measure LDPE biodegradation. Furthermore, the review addresses the challenges and opportunities associated with the implementation of these technologies in agriculture, as well as the organisms utilized for this purpose. The analysis presented in this manuscript can contribute to the awareness of the responsible use of plastics in the agricultural sector, to generate more sustainable and resilient agriculture in the context of global environmental challenges, while always prioritizing the welfare of human health.

## 2 Literature retrieval

The bibliographic review began with the search protocol, establishing the terms and criteria. The databases selected were: Scopus, Nature, Web of Science, and the Google Scholar search engine due to their recognition and high impact (Zárate-Rueda et al., 2021). The search equation [“Biodegradation” OR “Biological degradation” OR “Biomineralization” AND (“Low-density polyethylene OR LDPE”)] was applied and the filters of the scientific tools were used with the application of inclusion and exclusion criteria. The inclusion criteria were keywords such as “Polyethylene” and “Biodegradation,” in original research articles and conference papers, published in the period from 2019 to 2024, in the English language. Subsequently, a review of article titles and abstracts was performed. A total of 118 articles were obtained, of these 116 original research articles and 2 conference articles. Those articles that aligned with the objectives of the present study were selected and managed in the free license software Mendeley desktop v1.19.8. These were downloaded, analyzed, and diagrammed using computer tools such as Microsoft Excel, Word, QGis, VOSviewer, and Python as programming languages through the: Os, Pandas, Matplotlib, Seaborn, WordCloud, and PyPDF2 libraries. To generate the distribution map of the studies that are part of the analysis of this manuscript, information on the year of publication, country of the institution of the first author was used, the base shape of the map was downloaded from the UN page and worked in the QGis v2.0.2 tool. Then the extracted information from the papers was analyzed and diagrammed, and finally, a work flowchart was proposed.

### 2.1 Geographical analysis

**Figure 2:**
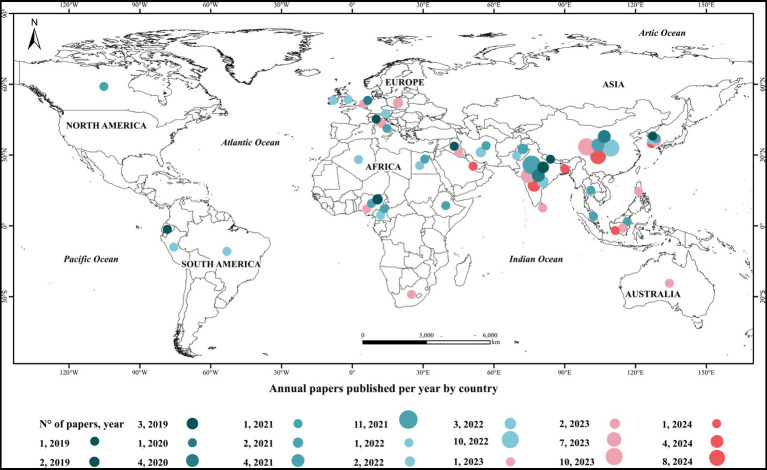
World distribution map of polyethylene biodegradation articles published from 2019 to 2024.

The geographic distribution of articles published between 2019 and 2024, and focused on the biodegradation of plastic used in agriculture are presented in Figure 2. The data indicate a significant concentration of studies in Asia, particularly in China and India, which together account for 68 studies; these countries with the highest number of research also stand out for their substantial production of plastics. Between May 2021 to May 2024 alone, China produced 6.52 million metric tons of plastic products (Statista, 2024a). Similar is the case with India, which in 2022 produced a volume of 1.7 million metric tons of plastics (Statista, 2024b). Continents such as Oceania and South America present few studies related to the subject, while Oceania presents only one study during the period analyzed. This disparity highlights the low commitment to research on the biodegradation of plastics and underlines the need for a broader commitment to address plastic pollution fully. In Europe, a more balanced distribution of studies is observed, with countries such as Germany, Spain, and the UK contributing notably (Figure 2). This trend may be related to stringent environmental policies and research funding in the European Union, which promotes sustainable solutions for plastic waste management. In Canada, research activity is evident, aligning with its national policies to reduce plastic waste and promote a circular economy (CEPA, 1999). In addition, the map in Figure 2 reveals a growing contribution from developing countries, particularly in Africa and Latin America. In this case, countries such as Brazil and South Africa are beginning to emerge in the field of plastic biodegradation research, possibly driven by the need to manage large amounts of plastic waste and the implementation of stricter environmental policies; they also receive foreign funding as part of biodiversity conservation efforts driven by plastic-producing countries.

The uneven distribution of research also reflects differences in the technological capacity and resources available for research in different regions. While developed countries have access to more funding and advanced technologies (Hsu et al., 2022), developing countries face challenges related to infrastructure and funding, which limits their ability to conduct high-quality research (Orona-Návar et al., 2022). In this context, it is essential to continue fostering international collaboration to share knowledge and strengthen human talent and technologies that can accelerate progress in plastics biodegradation research. Cooperative programs and joint funding can help to balance regional management inequalities and promote global solutions to plastic pollution.

### 2.2 Keywords

**Figure 3:**
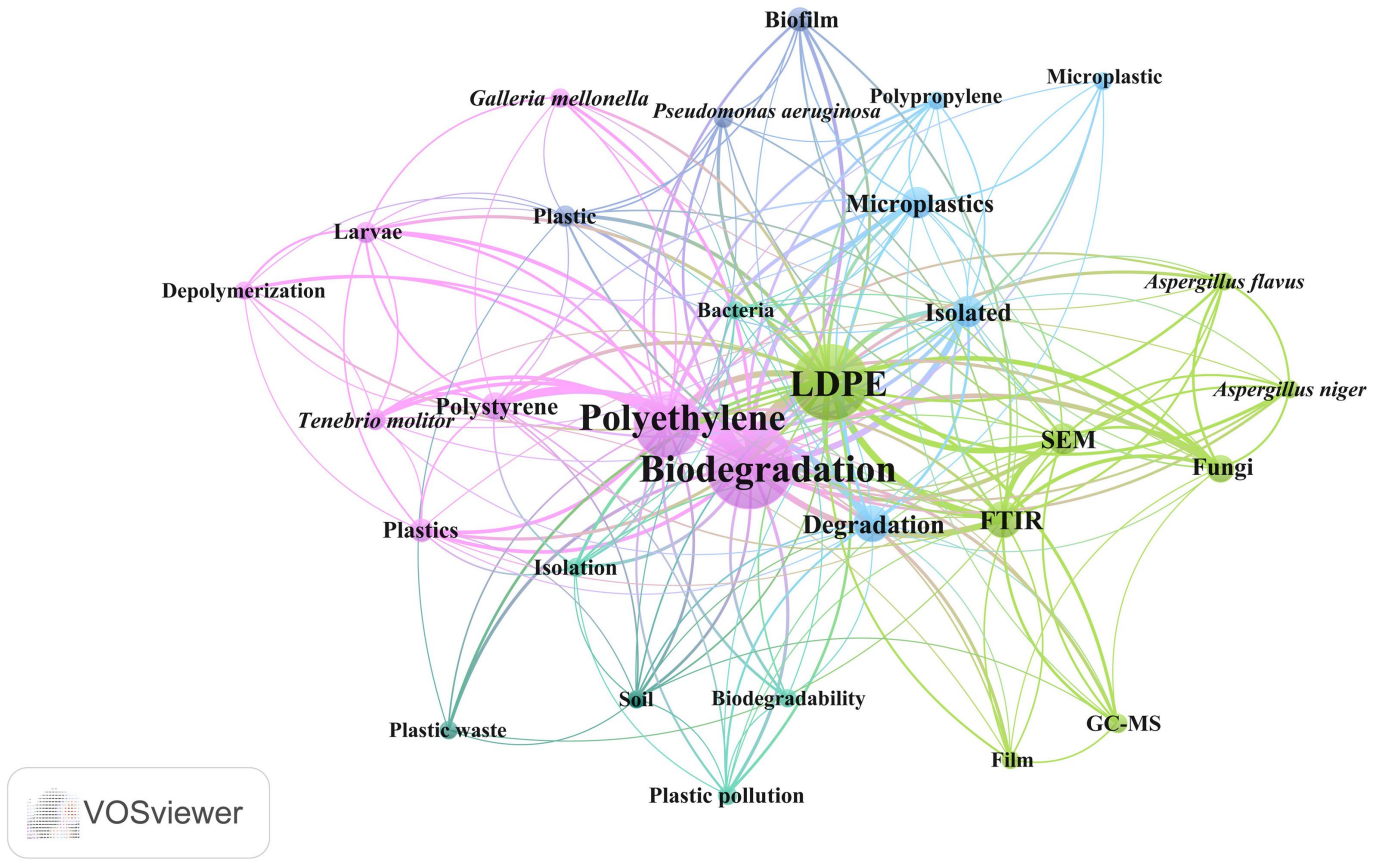
Network map for keywords and titles associated with LDPE biodegradation.

The graph generated in VOSviewer represents a co-occurrence map of terms used in titles and keywords related to the biodegradation of LDPE and other plastics in articles published between 2019 and 2024. The largest and most central nodes, such as “Biodegradation,” “Polyethylene” and “LDPE,” indicate that these terms are the most mentioned and relevant in the analyzed articles. The connections between the nodes reflect the frequency with which these terms appear together in the same studies, suggesting a strong interrelationship between the concepts. Likewise, terms in the green cluster, such as “FTIR,” “GC–MS,” “SEM” and “Fungi,” indicate a focus on analytical techniques and organisms associated with biodegradation. Their prominence is related to methodologies used to assess plastic degradation after various treatments. The blue cluster, which includes terms such as “Microplastics,” “Polypropylene” and “Isolated,” highlights research focused on the degradation of different plastics and microorganisms. The pink group, which contains terms such as “Polystyrene,” “*Tenebrio molitor*” and “Depolymerization,” reflects an interest in the degradation of specific plastics and associated degradation mechanisms (Figure 3).

## 3 Industrial biodegradation

Industrial biodegradation offers a critical solution for managing polyethylene (PE) waste by utilizing controlled environments to optimize the activity of microbial consortia and enzymatic systems. This approach capitalizes on advanced methodologies to address the limitations of natural biodegradation in uncontrolled settings. Constant developments highlight the use of enzyme-facilitated processes, such as those employing oxidoreductases and laccases, which break down complex polymers into simpler, more manageable compounds. These enzymes, often derived from marine microorganisms, exhibit remarkable stability under industrial conditions, including variable temperatures, pH, and salinity, making them highly suitable for large-scale applications (Ayilara and Babalola, 2023; Sivaperumal et al., 2017). Additionally, specialized bioreactors have been developed to enhance microbial activity, ensuring consistent degradation rates and efficient conversion of plastic into non-toxic byproducts, such as carbon dioxide and water (Verma and Jaiswal, 2016). The effectiveness of industrial biodegradation is further amplified by leveraging microbial consortia capable of synergistic actions. Bacterial and fungal strains have demonstrated the ability to depolymerize PE and similar materials, transforming them into oligomers that are subsequently mineralized. The controlled nature of industrial processes also allows for the use of genetically modified organisms, tailored to target specific types of plastic waste, thereby maximizing efficiency and minimizing residual contamination. Moreover, advancements in bioprocess technology, including the integration of co-metabolism strategies, have shown significant promise in enhancing degradation rates, particularly for recalcitrant plastics (Rüthi et al., 2023).

## 4 Polyethylene biodegradation

**Figure 4:**
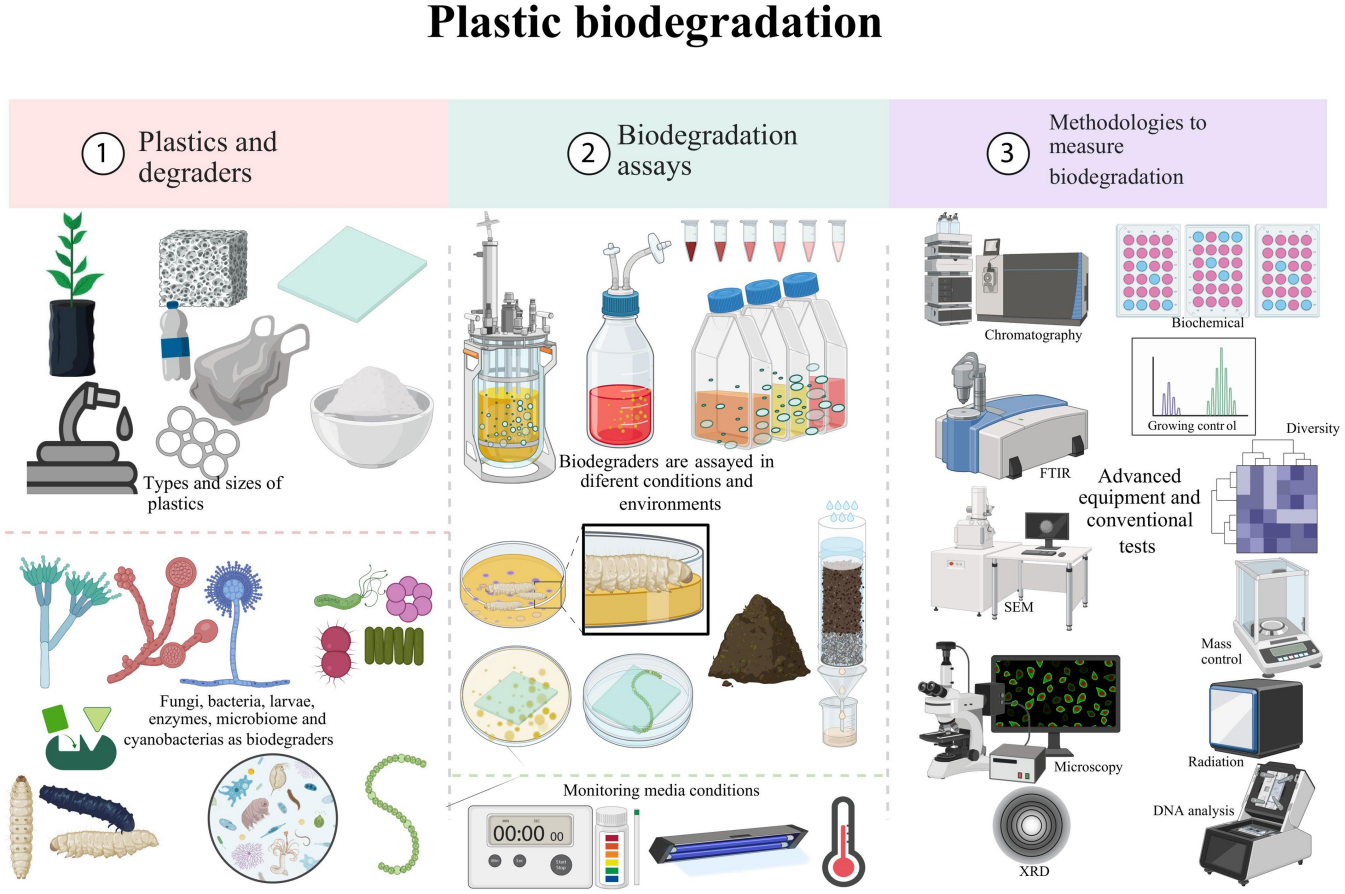
Graphical summarizing of the review.

In this section, the principal findings are described and summarized in Figure 4. In addition, the organization of data to make this section in Supplementary Table S1 which contains details of various organisms or substances and their origins, as well as corresponding pre-treatments, conditions, treatments, controls, evaluation times, and techniques used to measure degradation and identification. For each entry, the purpose of the techniques and the results are provided, with their references included for sources of data.

### 4.1 Organisms involved in the biodegradation of plastics

The degraders reported in the analysis period include bacteria (Table 1), fungi (Table 2), microalgae, microbial consortia, and insect larvae (Table 2), among others. Some of these microorganisms have been employed continuously while others only in specific years (Supplementary Figure S1). In the case of bacteria, they have been used consistently every year, reflecting their sustained effectiveness in plastic biodegradation. Fungi, although less frequent, have shown intermittent use, while microbial consortia, which combine bacteria and fungi, have recently gained interest because they combine the metabolic capabilities of several species to achieve more efficient biodegradation. These consortia can produce complementary enzymes that fragment plastic polymers faster and more effectively than individual microorganisms. Their ability to form biofilms improves the adhesion and decomposition of plastics, especially when their activity is stimulated with oxidizing agents such as H_2_O_2_, which enhances the production of key enzymes such as peroxidases (Skariyachan et al., 2021; Fachrul et al., 2024; Mohammadi et al., 2022) On the other hand, larvae have been occasionally employed, highlighting their potential in certain specific contexts (He et al., 2024; Burd et al., 2023; Yang et al., 2021) and algae (Sanniyasi et al., 2021) which represent an innovative strategy in LDPE biodegradation have also been used sporadically. This temporal distribution highlights the diversification of the methodologies applied to biodegradation in recent years, with an increasing trend toward the use of microbial combinations, as well as the exploration of new degraders such as larvae and algae.

**Table 1:** Report of bacteria studied in the biodegradation of LDPE and other plastics.

Plastic degrader bacteria	References
*Achromobacter denitrificans*	Maleki Rad et al. (2022)
*Achromobacter* sp.	Dey et al. (2020)
*Achromobacter xylosoxidans*	Tiwari et al. (2024)
*Acinetobacter sp.	Kunlere et al. (2019) and Yin et al. (2020)
*Acinetobactor calcoaceticus*	Pathak and Navneet (2023)
*Alcaligenes faecalis*	Nag et al. (2021) and Mohammadi et al. (2022)
*Alcanivorax* sp.	Zadjelovic et al. (2022) and Khandare et al. (2021)
*Bacillus albus*	Khan et al. (2021)
*Bacillus aryabhattai*	Montazer et al. (2021)
*Bacillus cereus*	Mishra et al. (2024), Maroof et al. (2021), Nanthini Devi et al. (2021), and Jebashalomi et al. (2024))
*Bacillus licheniformis*	Yao et al. (2022) and Rani et al. (2022)
*Bacillus paramycoides*	Nanthini Devi et al. (2021) and Wu et al. (2023)
*Bacillus siamensis*	Maroof et al. (2021)
*Bacillus* sp.	Mohy Eldin et al. (2022), Kunlere et al. (2019), Nanthini Devi et al. (2021), Ferrero et al. (2022), and Zhang et al. (2023)
*Bacillus* spp.	Kumar Gupta and Devi (2019), Fibriarti et al. (2021), and Kumar Shrestha et al. (2019)
*Bacillus subtilis*	Zahari et al. (2021), Mohammadi et al. (2022), Pathak and Navneet (2023), and Yao et al. (2022)
*Bacillus tropicus*	Samanta et al. (2020)
*Bacillus velezensis*	Liu et al. (2022)
*Bacillus wiedmannii*	Maroof et al. (2021)
*Clostridium sp.	Fachrul et al. (2024)
*Cobetia* sp.	Khandare et al. (2021)
*Enterococcus* sp.	Adithama et al. (2023)
*Exiguobacterium* sp.	Khandare et al. (2021)
*Gordonia polyisoprenivorans*	Wang et al. (2023) and Tiwari et al. (2023)
*Halomonas* sp.	Khandare et al. (2021)
*Halomonas venusta*	Dimassi et al. (2024)
*Klebsiella pneumoniae*	Wróbel et al. (2023) and Zhang et al. (2023)
*Kocuria rosea*	Mohammadi et al. (2022)
*Lysinibacillus fusiformis*	Mohammadi et al. (2022) and Montazer et al. (2021)
*Lysinibacillus sphaericus*	Mohammadi et al. (2022)
*Microbacterium steraromaticum*	Zhang et al. (2024)
*Microbacterium oxydans*	Montazer et al. (2021)
*Microbulbifer hydrolyticus*	Li et al. (2020)
*Oceanimonas* sp.	Joshi et al. (2022)
*Paenibacillus* sp.	Joshi et al. (2022)
*Paracoccus aminophilus*	Pathak and Navneet (2023)
*Pseudomona knackmussii*	Hou et al. (2022)
*Pseudomonas aeruginosa*	Tamnou et al. (2022), Gupta and Devi (2020), Pathak and Navneet (2023), Hou et al. (2022), Ferrero et al. (2022), Dimassi et al. (2024), and Mouafo Tamnou et al. (2021)
*Pseudomonas putida*	Pathak and Navneet (2023) and Ji et al. (2023)
*Pseudomonas* sp.	Kunlere et al. (2019) and Nadeem et al. (2021)
*Pulmonis* sp.	Tiwari et al. (2024)
*Raoultella* sp.	Yuan et al. (2023)
*Rheinheimera* sp.	Joshi et al. (2022)
*Rhodococcus opacus*	Zampolli et al. (2021) and Zampolli et al. (2023)
*Rhodococcus qingshengii*	Nie et al. (2021)
*Rhodococcus* sp.	Rong et al. (2024)
*Serratia marcescens	Wróbel et al. (2023) and Lou et al. (2022)
*Serratia* sp.	Nadeem et al. (2021)
*Shewanella* sp.	Joshi et al. (2022)
*Staphylococcus aureus*	Tamnou et al. (2022)
*Staphylococcus sp.	Kunlere et al. (2019)
*Stenotrophomonas maltophilia*	Adithama et al. (2023)
*Stenotrophomonas* sp.	Dey et al. (2020) and Nadeem et al. (2021)
*Streptomyces* spp.	Soud (2019)
*Thiobacillus sp.	Fachrul et al. (2024)
*Vibrio* sp.	Joshi et al. (2022)

*Indicates bacteria in consortia with other microorganisms, bacteria, or fungi.

**Table 2:** Report of fungi and larvae studied in the biodegradation of LDPE.

Organism	References
Fungi	
*Alternaria alternata*	Gao et al. (2022) and Aderiye et al. (2019)
*Aspergillus carbonarius*	El-Sayed et al. (2021)
*Aspergillus flavus	Obaid and AL-Jawhari (2023), Gong et al. (2023), Kunlere et al. (2019), Aderiye et al. (2019, DSouza et al. (2021), and Saira et al. (2022)
*Aspergillus fumigatus*	El-Sayed et al. (2021)
*Aspergillus niger*	Sáenz et al. (2019), Obaid and AL-Jawhari (2023), Gong et al. (2023), DSouza et al. (2021), and Saira et al. (2022)
*Aspergillus oryzae*	DSouza et al. (2021)
*Aspergillus* sp.	Aderiye et al. (2019)
*Aspergillus terreus	Sáenz et al. (2019) and Mohy Eldin et al. (2022)
*Candida tropicalis	Zahari et al. (2021)
*Cephalosporium* sp.	Chaudhary et al. (2023)
*Cladosporium sphaerospermum*	Sathiyabama et al. (2024)
*Collectotrichum fructicola*	Khruengsai et al. (2021)
*Dekkera sp.	Fachrul et al. (2024)
*Italian Diaporthe*	Khruengsai et al. (2021)
*Flavodon flavus*	Perera et al. (2023)
*Fusarium oxysporum*	Wróbel et al. (2023)
*Meyerozyma caribbica*	Elsamahy et al. (2023)
*Meyerozyma guilliermondii	Elsamahy et al. (2023) and Lou et al. (2022)
*Penicillium citrinum*	Khan et al. (2023)
*Penicillium simplicissimum*	Aderiye et al. (2019)
*Penicillium sp.	Mohy Eldin et al. (2022)
*Phlebiopsis flavidoalba*	Perera et al. (2023)
*Rhizopus* sp.	Harrat et al. (2022)
*Rhodotorula mucilaginosa*	Vaksmaa et al. (2023)
*Stagonosporopsis citrulli*	Khruengsai et al. (2021)
*Sterigmatomyces halophilus*	Elsamahy et al. (2023)
*Thermomyces lanuginosus*	Chaudhary et al. (2021)
*Thyrostroma jaczewskii*	Khruengsai et al. (2021)
*Trichoderma harzianum*	Bernat et al. (2023)
Larvae	
*Achroia grisella*	Ali et al. (2023)
*Corcyra cephalonica*	Soleimani et al. (2021)
*Galleria mellonella*	Burd et al. (2023), Kundungal et al. (2021), Lou et al. (2021), and Poma et al. (2022)
*Galleria mellonella*	Sanluis-Verdes et al. (2022)
*Hermetia illucens*	Wang et al. (2024)
*Tenebrio molitor*	He et al. (2024), Yang et al. (2021), Wang et al. (2022), Yang et al. (2021), Peng et al. (2023); Lou et al. (2021), and Yang et al. (2022)
*Tenebrio obscurus*	Yang et al. (2021) and Yang et al. (2022)
*Zophobas atratus*	Wang et al. (2022), Zaman et al. (2024), Peng et al. (2022), and Peng et al. (2020)
*Zophobas morio*	Wang et al. (2022)

*Indicates fungi in consortia with other microorganisms, other fungi, or bacteria.

### 4.2 Comparison between different agents

Studies on LDPE biodegradation demonstrate varying efficiencies depending on the organism and conditions. Weight loss percentages range from 1.50 to 88.50%, with *Zophobas atratus* larvae achieving the highest reduction (88.50% in 45 days) (Wang et al., 2022). Fungi such as *Aspergillus niger* (Sáenz et al., 2019), and bacteria like *Alcaligenes faecalis* (Nag et al., 2021), also show promising results, with weight loss exceeding 20% in specific setups. These results highlight the diverse potential of biological agents in plastic degradation and the differences in percentages of weight loss (Table 3).

**Table 3:** Polyethylene biodegradation studies and its performance.

Organism	Type of degrader	Shape and size	Evaluation time*	Results—weight loss report as %	References
*Cladosporium sphaerospermum*	Fungus	LDPE films (2×2 cm, 7 mg)	7 days	15.12%	Sathiyabama et al. (2024)
*Rhizopus* sp.	Fungus	LDPE films (0.4 g)	30 days	20%	Harrat et al. (2022)
*Thermomyces lanuginosus*	Fungus	LDPE films (8 μm thick, 4×4 cm)	30 days	9.21 ± 0.84%	Chaudhary et al. (2021)
*Pseudomonas aeruginosa*	Bacterium	LDPE film (4×4 cm)	30 days	6.25%	Mouafo Tamnou et al. (2021)
*Klebsiella pneumoniae*	Bacterium	LDPE film (4×4 cm)	30 days	2.21%	Zhang et al. (2023)
*Bacillus tropicus*	Bacterium	LDPE films (10 μm thickness)	40 days	10.15%	Samanta et al. (2020)
*Bacillus cereus*	Bacterium	LDPE film 3×3 cm	42 days	4.13 ± 0.81%	Jebashalomi et al. (2024)
*Pseudomonas* sp. *Acinetobacter* sp. *Bacillus* sp. *Aspergillus flavus*	Bacterial-Fungal Consortium	LDPE granules (0.4 g)	42 days	3.75%	Sarmah and Rout (2019)
*Nostoc carneum*	Cyanobacteria	LDPE strips (1 × 1 cm, 20 μm thickness)	42 days	27%	Sarmah and Rout (2019)
*Zophobas atratus*	Larva	PE foam (8 × 5 × 2 cm pieces)	45 days	88.50%	Wang et al. (2022)
*Cephalosporium* sp.	Fungus	LDPE films (4×4 cm, 69 μm thickness)	56 days	24.53% ± 0.73%	Chaudhary et al. (2023)
*Bacillus* spp.	Bacterium	LDPE films (3 × 3 cm)	60 days	1.50%	Kumar Gupta and Devi (2019)
*Zophobas atratus*	Larva	LDPE sheets	60 days	24.04%	Zaman et al. (2024)
*Alcaligenes faecalis*	Bacterium	PE strips (2×2 cm, 28 mg for white)	70 days	21.72 ± 2.1%	Nag et al. (2021)
*Aspergillus niger*	Fungi	LDPE films (squares of 2 cm^2^)	77 days	35.30%	Sáenz et al. (2019)
*Aspergillus terreus*	Fungi	LDPE films (squares of 2 cm^2^)	77 days	22.14%	Sáenz et al. (2019)
*Stenotrophomonas maltophilia*	Bacteria	LDPE beeads	90 days	5%	Adithama et al. (2023)
*Enterococcus* sp.	Bacterium	LDPE beeads	90 days	6%	Adithama et al. (2023)
*Penicillium citrinum*	Fungus	LDPE films (4×3 cm)	90 days	38.82%	Khan et al. (2023)
*Achromobacter denitrificans*	Bacterium	LDPE microplastics (250–425 μm)	90 days	6.50%	Maleki Rad et al. (2022)
*Bacillus siamensis*	Bacterium	LDPE films, 2 cm × 2 cm, 230 micron thickness	90 days	8.46 ± 0.3%	Maroof et al. (2021)
*Bacillus cereus*	Bacterium	LDPE films, 2 cm × 2 cm, 230 micron thickness	90 days	6.33 ± 0.2%	Maroof et al. (2021)
*Bacillus* sp.	Bacterium	PE bags	105 days	3.0%	Mohy Eldin et al. (2022)
*Penicillium* sp.	Fungus	PE bags	105 days	2.70%	Mohy Eldin et al. (2022)
*Aspergillus terreus*	Fungus	PE bags	105 days	6.60%	Mohy Eldin et al. (2022)
*Aspergillus carbonarius*	Fungi	LDPE sheets (2 cm × 2 cm)	112 days	3.80%	El-Sayed et al. (2021)
*Aspergillus fumigatus*	Fungi	LDPE sheets (2 cm × 2 cm)	112 days	2.27%	El-Sayed et al. (2021)
Consortium	Fungi consortia	LDPE sheets (2 cm × 2 cm)	112 days	5.01%	El-Sayed et al. (2021)

*Table organized by evaluation time from shorter to largest.

Each type of organism has unique degradation capabilities that are influenced by its origin, environmental conditions, and previous treatments. Table 4 presents some advantages and disadvantages of the different organisms in the process of biodegradation of plastics. In the case of degrading bacteria, they commonly belong to genera such as *Pseudomonas, Bacillus, Streptomyces* and *Rhodococcus. Pseudomonas putida* (Li et al., 2024), is known for its ability to degrade polyesters, whereas *Bacillus subtilis* (Kumar Gupta and Devi, 2019), has shown successful degradation of polystyrene. Fungi have a significant advantage due to their ability to secrete large amounts of enzymes such as; peroxidase, and lignin peroxidase enzymes outside their cells, which allows the degradation of complex polymers in the immediate environment (Wang et al., 2019); this characteristic has enabled multiple assays in plastic degradation. One example is *Rhodotorula mucilaginosa*, a fungus isolated from marine sediments.

**Table 4:** Comparison of types of plastic-degrading organisms.

Type of degrader	Advantages	Disadvantages
Bacteria	- Wide diversity and adaptability to different environments. - Ability to degrade a variety of plastics, including LDPE and polyesters. - High growth rate. - Simple cultivation in liquid or solid media.	- Degradation limited to specific polymers. - Dependence on optimal environmental conditions for maximum efficiency. - Reduced capacity to secrete extracellular enzymes. - Quantification requires precise molecular techniques.
Mushrooms	- Production of extracellular enzymes that facilitate the degradation of complex polymers. - Ability to degrade various plastics, such as PE, PU, and PP. - Resilience in extreme environments. - They can be grown on solid or liquid substrates, including organic waste.	- Slower growth rate than bacteria. - They require specific humidity and temperature conditions. - They can be adversely affected by the presence of contaminants. - Quantification can be more complex and requires enzymatic assays.
Microalgae	- They can generate compounds that facilitate the biodegradation of plastics. - Integration with wastewater treatment systems. - Cultivation in open or closed systems, with a high biomass production rate.	- Limited direct degradation capacity compared to bacteria and fungi. - They require specific light and nutrient conditions. - Quantification involves measuring the growth and production of specific metabolites.
Microbial consortia	- Synergy between different microorganisms that can increase degradation efficiency. - Ability to degrade a wider variety of plastics simultaneously. - They can be grown in bioreactors or fermentation systems.	- Complexity in the management and control of consortia. - They require specific environments that favor cooperation between species. - Quantification requires analysis of population and metabolite dynamics.
Larvae	- Ability to degrade plastics by digestion, combining mechanical and enzymatic action. - Possibility of integration into sustainable agricultural systems. - Relatively simple breeding and management in certain species.	- Large numbers of larvae are required to degrade significant volumes of plastic. - Environmental factors critical to their survival. - Quantification includes weight tracking and residue analysis.

In recent studies, this fungus was exposed to polyethylene treated with ultraviolet (UV) radiation, a pretreatment that favors plastic biodegradation (Giyahchi and Moghimi, 2023). Therefore, the ability of fungi to degrade plastics is not limited to only *R. mucilaginosa* species, but also to other fungi, such as the genera *Aspergillus, Penicillium,* and *Fusarium*, as they have also shown potential in the biodegradation of different types of plastics. For example, *Aspergillus niger* (Fachrul et al., 2024), has been reported for its ability to degrade polyurethane, while *Penicillium chrysogenum* (Khan et al., 2023), and *Fusarium solani* (Wróbel et al., 2023), have shown effectiveness in the degradation of polyethylene and polypropylene. Certain microorganisms can produce compounds that facilitate the decomposition of plastic by other organisms in the consortium, this is due to the release of compounds such as organic acids, peroxides, and other secondary metabolites (Tiwari et al., 2024; Wang et al., 2023), which modify the structure of the plastic and make it more susceptible to enzymatic degradation.

As for the enzymes, laccase, peroxidase, and cutinase (Gao et al., 2022; Tamnou et al., 2022), are some of the most studied in this context. These enzymes can break the chemical bonds of plastic polymers, facilitating their decomposition into monomers and other simpler compounds, such as carbon and carbon dioxide (CO_2_). Often, this material is integrated into the organism as part of its carbon source. However, the production of these enzymes may be limited by factors such as nutrient availability, pH, temperature, and the presence of specific inducers, such as plastid-related substrates and cofactors necessary for enzymatic activity (Khandare et al., 2022; Li et al., 2023).

The biodegradation of plastics using larvae has emerged as a novel and promising approach. Various insect species, in particular the larvae of certain beetles and flies, have demonstrated the ability to degrade plastics through their digestive processes. This approach not only offers an environmentally friendly alternative for plastic waste management but can also be integrated with sustainable agricultural practices. One documented case is the beetle *Tenebrio molitor*, commonly known as the mealworm. Larvae of *T. molitor* can degrade plastics such as PS and PE (Wang et al., 2023; Wang et al., 2024). This process is facilitated by the presence of symbiotic microorganisms in the larval digestive system, which produces enzymes such as alkane monooxygenase (Zaman et al., 2024), and laccase (Kundungal et al., 2021), that are capable of breaking down these polymers into smaller carbon chains and sometimes into CO_2_ and water. *T. molitor* larvae can consume approximately 34–39 mg of EPS, a plastic widely used in containers and packaging (Wang et al., 2022). As a result, EPS is oxidized and fragmented, with a significant reduction in its molecular weight. However, many times plastic can be integrated into the same organism of larvae in the form of microplastics and nanoplastics, and although it may seem insignificant this can be a problem since the end of these larvae can be food for other larger organisms and indirectly can expand and integrate into their organism (Yang et al., 2021).

Another species, that has shown potential in the biodegradation of plastics, is the black soldier fly (*Hermetia illucens*). The larvae of *H. illucens* are known for their ability to break down organic matter and have been studied for their ability to degrade bioplastics such as polyhydroxyalkanoate (PHA) and polylactate (PLA), breaking down PLA into simpler compounds such as lactic acid and CO_2_ (Wang et al., 2024). Therefore, this helps us to understand that the efficiency of larvae in biodegrading plastics is enhanced by the synergy between insects and the microorganisms present in their gut. Symbiotic bacteria and fungi produce a variety of hydrolytic enzymes, such as esterase, cutinase, and lipase, which are essential for the degradation of plastic polymers. In addition, the mechanical action of chewing and the intestinal movement of the larvae facilitates the fragmentation of the plastic, increasing the surface area exposed to enzymatic action, this process although not a decomposition is an important part of subsequent biodegradation processes (Ma et al., 2023).

The integration of plastic-degrading larvae in agricultural systems has multiple benefits. Not only does it contribute to reducing plastic waste in the agricultural environment, but it also produces valuable by-products such as frass, the so-called “insect excrement,” which can be used as an organic fertilizer. The resulting frass is applied to crops as fertilizer, and although the mature larvae can be fed for poultry or fish, creating a more sustainable and circular agricultural system, (Peng et al., 2022). However, the effect of possible micro and nano plastics on larvae should be studied in detail. Therefore, an integrated system model in on-farm plastic waste management could include the use of *T. molitor* larvae, where agricultural plastics such as PE mulch can be degraded *in situ*. Despite the promising potential, using larvae for biodegradation of plastics faces several challenges. One of the main ones is optimizing culture conditions to maximize degradation efficiency. In addition, it is necessary to ensure that micro-and nanoplastics do not enter the food chain, due to their negative effects such as drug resistance and new diseases (Shi et al., 2024).

Supplementary Figure S2 presents a comparative analysis of the average evaluation time for different organisms or substances used in the biodegradation of polyethylene. A great variability in the evaluation times is observed, which reflects both the diversity of the organisms studied and the complexity of the biodegradation processes. Some microorganisms such as *Brevibacillus brevis* (Tiwari et al., 2023), and the soil microbiome (Ma et al., 2023; Jayan et al., 2023; Maddison et al., 2023; Peng et al., 2023; Yuan et al., 2023; Zhang et al., 2023), stand out as having significantly long evaluation times, exceeding 450 days. This suggests that these microorganisms may have a slow biodegradation process, or that studies conducted with them have required a prolonged time to observe significant results. In contrast, the fungus *Rhizopus* sp. (Gupta and Devi, 2020), and the beetle *Zophobas atratus* (He et al., 2024; Yang et al., 2021; Lou et al., 2021; Sanluis-Verdes et al., 2022), show evaluation times in the range of 20–60 days, which could indicate higher biodegradation efficiency or studies designed for faster results. Different strains of *Z. atratus* and their combinations with other organisms such as *Tenebrio molitor* show a wide range of evaluation times, suggesting that the interaction between species and the specificity of the organisms used have a significant impact on biodegradation efficiency and the conditions they require.

Another notable aspect is the case of the bacterium *Gordonia polyisoprenivorans* (Wang et al., 2023), which has an intermediate evaluation time, which could be related to its specialized capabilities in the degradation of complex polymers but requires considerable time to demonstrate its effectiveness. The use of microbial consortia, such as Bacterial-Fungal Consortium (BFC) (Li et al., 2024; Adithama et al., 2023; Bao et al., 2023; Gong et al., 2023), seems to offer a robust strategy, with more efficient assessment times compared to some individual microorganisms, suggesting that cooperation between different types of microorganisms could accelerate the biodegradation process, so the outlook for such studies is encouraging, although interactions between different taxonomic groups could result in antagonism and competition, requiring careful observations to create partnerships that can work successfully. A deducible rule of thumb is that more specialized organisms, such as certain fungi and bacteria, tend to require less time for evaluation, whereas microbial consortia and microorganisms working in more complex environments may need longer times to show results. However, this may also indicate that, in the future, the development of optimized consortia and genetic engineering techniques could significantly reduce these times, making biodegradation faster and more efficient. It is worth mentioning that for practicality a single study that evaluated Soil microbiome and climatic conditions for 2,556 days was not represented in Supplementary Figure S2, more details in Supplementary Table S1.

## 5 Aspects of LDPE in biodegradation processes

### 5.1 Mechanism of biodegradation

Microorganisms, including bacteria and fungi, attach to the polymer surface using biofilm-forming mechanisms and secrete compounds like exopolysaccharides and surfactants to further degrade the structure (Sáenz et al., 2019; Elsamahy et al., 2023). During biofragmentation, extracellular enzymes such as hydrolases, laccases, and esterases break down long polymer chains into smaller oligomers and monomers (Li et al., 2024). The efficiency of this stage depends on the chemical composition, crystallinity, and hydrophobicity of the polymers, as well as the availability of cofactors like oxygen and nutrients. Once fragmented, the smaller compounds are taken up by microbial cells in the assimilation stage, where they undergo intracellular metabolic processes to produce energy, biomass, and intermediate metabolites (He et al., 2024). Factors like microbial metabolic diversity, the availability of electron donors and acceptors, and community interactions significantly influence this stage (Sunny and Saleena, 2020). The final phase, mineralization, involves the complete degradation of organic matter into inorganic molecules such as carbon dioxide, water, and methane (El-Sayed et al., 2021; Zahari et al., 2021; Khan et al., 2023). This stage is heavily influenced by environmental parameters, including pH, redox potential, and oxygen availability, which dictate whether the process occurs under aerobic or anaerobic conditions (Obaid and AL-Jawhari, 2023). In addition to these environmental factors, microbial interactions play a critical role in enhancing biodegradation efficiency. Cooperative relationships, such as cross-feeding and enzyme complementation, improve substrate utilization, while competitive interactions can reduce efficiency by limiting resource availability (Pinto et al., 2022). Operational parameters, such as temperature, moisture content, agitation, and nutrient supplementation, are also key drivers of biodegradation outcomes. These factors, extensively studied in the literature, underline the complexity of the process, demonstrating how microbial and environmental conditions synergistically dictate the transformation of polymers into simpler, environmentally benign products, such as carbon dioxide, water, and biomass (Temporiti et al., 2022; Thomas Zumstein et al., 2018; He et al., 2024; Figure 1).

### 5.2 Parameters affecting biodegradation efficiency

The parameters influencing biodegradation efficiency in the reviewed studies are diverse and often interdependent, highlighting the complexity of this process. Key variables include microbial interactions, operating conditions, and substrate-specific characteristics. For instance, *Rhodotorula mucilaginosa* demonstrated varied efficiency depending on the UV pre-treatment duration and the addition of 13C-PE as a sole carbon source (Vaksmaa et al., 2023). Similarly, the inclusion of enriched media, such as mineral salt broth inoculated with *Gordonia polyisoprenivorans*, enhanced LDPE degradation over 35 days, emphasizing the role of nutrient availability and sterilization methods (Tiwari et al., 2023) incorporating microbial consortia, such as *Bacillus licheniformis* and *Achromobacter xylosoxidans*, further underscore the significance of synergistic microbial interactions, which enhance degradation pathways (Fachrul et al., 2024). Environmental conditions also critically impact biodegradation outcomes. Temperature, pH, and agitation significantly influenced results, as seen with *Bacillus subtilis*, which required optimized incubation parameters to achieve notable LDPE degradation (Mohammadi et al., 2022). Furthermore, pre-treatment processes, such as UV irradiation or chemical oxidation, were consistently noted to enhance microbial attachment and enzymatic activity, as demonstrated in *Microbulbifer hydrolyticus* experiments with LLDPE particles (Li et al., 2020), in the S1 the columns “Organism or substance and its origin,” “Type of degrader,” “Pre-treatment condition and initial size,” “Treatments,” and “Evaluation Time” can give an extent analysis about this point.

### 5.3 Forms of LDPE undergoing biodegradation

In LDPE biodegradation studies, the physical form of the plastic plays an important role in the interaction with the degrading organisms. The different presentations of the plastic, such as films, powder, sheets, beads, granules, and foams, among others (Supplementary Table S2), affect the surface area available for adhesion and subsequent degradation. In the publications analyzed, 46% used plastics in the form of films (Supplementary Figure S3), this form offers a larger surface area in contact, which favors adhesion and colonization (Skariyachan et al., 2021; Huang et al., 2022; Jeon et al., 2021; Kong et al., 2024; Puglisi et al., 2019). Their advantage lies in their similarity to agricultural plastics, such as mulching films, making them relevant models in these studies. Their disadvantage is their thickness and low resistance to aggressive pretreatment, which may disintegrate them before completing biodegradation. The plastic powder was 18.4%, and the reduced particle size positively influences the rapid interaction with microorganisms (Tiwari et al., 2023; Bernat et al., 2023; Montazer et al., 2021; Pathak and Navneet, 2023; Yang et al., 2022). Their limitation is the additional mechanical pretreatment for their production, hindering complexity in the practical application of the biodegradation process in the agricultural sector. 16.1% report the use of sheets, which are more manageable and have greater durability compared to pretreatments. In some cases, they may take longer to degrade due to their thickness (Pinto et al., 2022; Zaman et al., 2024; Yuan et al., 2023; Hou et al., 2022; Joshi et al., 2022; Mishra et al., 2024), Bags with 6.9% represent a high surface-to-volume ratio, which favors biodegradation. The main difficulty of this form is the uniformity of the experimental conditions and their respective comparisons with other studies, taking into account the different sizes and thicknesses (Jayan et al., 2023; Gerritse et al., 2020; Mohy Eldin et al., 2022; Nademo et al., 2023; Nnaji et al., 2021; Poma et al., 2022) LDPE foams with 5.75% present a porous structure that increases the interaction with microorganisms, allowing better penetration of nutrients and microbial enzymes, and accelerating the biodegradation process. Its limitation is the difficulty of the precise quantification of the degraded material (Yang et al., 2021; Peng et al., 2022; Lou et al., 2021; Wang et al., 2022), Granules (4.6%) and beads (2.3%), being more compact in shape, reducing the surface area available for microbial colonization, reducing biodegradation efficiency. However, as they are easy to manipulate and quantify, they are useful in small-scale studies (Khandare et al., 2022; Adithama et al., 2023; Dey et al., 2020; Kunlere et al., 2019; Zadjelovic et al., 2022).

### 5.4 Pretreatment of LDPE under biodegradation

Most studies report the pretreatment of the plastic in the biodegradation process, taking into account that this increases its susceptibility to microbial degradation. One of the most common treatments is exposure to UV radiation, a process that partially degrades the plastic, facilitating its decomposition by modifying its physical and chemical properties (Yuan et al., 2023; Chaudhary et al., 2023; Min et al., 2020; Montazer et al., 2020). To mention an example, LDPE treated with UV radiation for 48 h develops carbonyl groups and peroxides on its surface, which enhances the adhesion and activity of bacteria isolated from landfills, resulting in higher production of degrading enzymes and a higher biodegradation rate compared to untreated plastics (Soleimani et al., 2021). In the case of polyester, chemical treatment with nitric acid facilitates its decomposition by bacteria of the genus *Pseudomonas*. Culture conditions also play a key role in biodegradation, as factors such as optimum temperature (30°C for HDPE), pH (7.0 for polystyrene), and the availability of nutrients, such as glucose, influence microbial activity (Ji et al., 2023).

### 5.5 Growing conditions

By using plastic as the sole or main carbon source, a selective pressure is induced that forces the organism to consume the polymer, which allows observing its behavior and evolution under these challenging conditions. Culture conditions, such as temperature, pH, and nutrient availability, are critical factors affecting microbial activity and the efficiency of plastic biodegradation. Most studies maintain controlled conditions to optimize the activity of the degrading organisms, varying these conditions according to the type of organism and plastic to be degraded. Aderiye et al. (2019), in their study published in 2023 found that the optimum temperature for the biodegradation of HDPE by bacteria isolated from a waste landfill was 30°C. On the other hand, Chaudhary et al. (2023), found that a pH of 7 was optimal for the enzymatic activity of *Pseudomonas fluorescens* in the biodegradation of polystyrene. DSouza et al. (2021), showed that the addition of glucose in the experimental setups as an additional carbon source increased the rate of polypropylene biodegradation by *Aspergillus niger*. Oxygen concentration is another crucial factor that can influence the biodegradation of plastics. In one study, bacteria of the genus *Bacillus* were found to be more effective in the degradation of polyethylene under aerobic conditions, while bacteria of the genus *Clostridium* showed higher efficiency under anaerobic conditions (Islami et al., 2019; Fibriarti et al., 2021).

### 5.6 Limitations and considerations in the biodegradation of polyethylene in the agricultural system

Biodegradation of polyethylene in agricultural systems faces limitations due to the chemical nature of this polymer and the specific conditions of agricultural environments. PE, widely used in agricultural plastics such as mulching and coverings, has a molecular structure with highly stable carbon–carbon bonds, which makes its biological decomposition difficult (Hou et al., 2022). In these systems, soil organisms, such as larvae, and microorganisms such as fungi and bacteria, must compete with other more accessible carbon sources, which reduces the metabolic priority of PE and slows down its biodegradation (Yang et al., 2021; Montazer et al., 2020). Environmental factors such as temperature, pH, humidity, and nutrient availability vary considerably in the soil, limiting the enzymatic activity necessary for polymer decomposition (Montazer et al., 2020). In many cases, PE requires pretreatments to increase its bioavailability to microorganisms. Exposure to UV radiation or the use of oxidizing agents can partially break the polymer chains, increasing the susceptibility of the plastic to biodegradation (Yuan et al., 2023). However, these pretreatments not only increase operational costs but can also generate byproducts that alter soil microbial dynamics or negatively impact the quality of the agricultural ecosystem. Smaller fragments, such as microplastics generated by partial degradation of mulch, can be integrated into the soil, which presents a double challenge: on the one hand, they increase the contact surface for microorganisms, but on the other, they pose ecological risks by being transported to other parts of the ecosystem and incorporated into food chains. Intermediate products generated during biodegradation, such as low molecular weight compounds, must be assessed at a toxicological level, to ensure that they do not interfere with soil health or crops (Nelson et al., 2022).

## 6 Biodegradation measurement techniques

Table 5 presents a summary of the main techniques used in the measurement of biodegradation of plastics, describing the purpose and expected results of each. These techniques range from FTIR, which can identify changes in the functional groups and chemical structure of the material, to SEM, which focuses on observing physical changes and the formation of biofilms on the surface of plastics. In addition, techniques such as EDS and XPS are used to analyze elemental composition and chemical properties, while TGA and DSC evaluate the thermal stability of materials undergoing biodegradation. HPLC and GC–MS allow the identification of specific degradation products, such as organic acids and complex compounds, while the use of XRD and PY-GC–MS focuses on changes in crystallinity and differences in the chemical composition of treated polymers. The evolution of CO_2_ is also measured to assess the amount of gas released as an indicator of biodegradation, carbon conversion, and mineralization.

**Table 5:** Techniques most commonly used in the measurement of biodegradation of plastics.

Technique	Purpose	Expected results
FTIR (Fourier transform infrared spectroscopy)	Identify changes in functional groups and chemical structure of the material.	Formation of new functional groups such as carbonyl (C=O) and hydroxyl (OH) in the treated plastics, indicating. Changes in the existing functional groups such as reduction in the peaks associated with C-H and C-C bonds. An increase in the carbonyl index indicates the formation of new carbonyl groups due to oxidation and degradation of the material.
SEM (Scanning Electron Microscopy)	Observe structural changes Study biofilm formation	Cavity and void formation Increased surface roughness Erosion, cracking, and other surface damage Biofilm formation
EDS (energy dispersive X-ray spectroscopy)	Analyze elemental composition changes	Decrease in carbon content
Gravimetric Analysis	Measuring the mass difference	Loss of material weight
XPS (X-Ray Excited Photon Spectroscopy)	Evaluating chemical composition and surface properties	Increase in oxygen concentration the appearance of peaks representing ether bonds and carboxyl groups in the C1s scan Increase in the ratio of-C-H-bonds and strengthening of the-C-O-group Presence of new functional groups.
DRX (X-Ray Diffraction)	Determine the changes in crystallinity and phase structure of the treated materials.	Decrease in crystallinity Crystallinity index variations Structural changes
TGA (Thermogravimetry) and DSC (Differential Scanning Calorimetry)	Evaluating thermal stability Measuring changes in thermal properties	Reduction in thermal stability Changes in the decomposition temperature
HPLC (High-Performance Liquid Chromatography)	Identify plastic degradation products	Identification of butyrate
UP-HPLC (Ultra Performance Liquid Chromatography)	Detailed analysis of organic acids and biodegradation products with high resolution.	Identification of organic acids such as citric, malic, and oxalic acid. Degradation products such as dodecane
CO_2_	Measure the amount of CO released	Increase in CO_2_
GC–MS (Gas Chromatography–Mass Spectrometry)	Identification of degraded products and metabolites	Identification of compounds such as bis(2-ethylhexyl) phthalate, 2-propenoic acid, tetradecyl ester, tetracontan in the degradation of LDPE, alkane, alcohols, ketones, acids, and other biodegradation products.
GC–MS/MS (Gas Chromatography—Tandem Mass Spectrometry with Mass Spectrometry)	More accurate identification and quantification of degradation products.	
GC–MS with MSD (Gas Chromatography–Mass Spectrometry with Scattering Mass Detectors)	Analysis of degradation products and metabolites during the growth of plastics	
PY-GC–MS (Pyrolysis—Gas Chromatography–Mass Spectrometry)	Determination of chemical composition differences between original samples and polymer residues.	

## 7 Considerations for control of biodegradation test results

**Figure 5:**
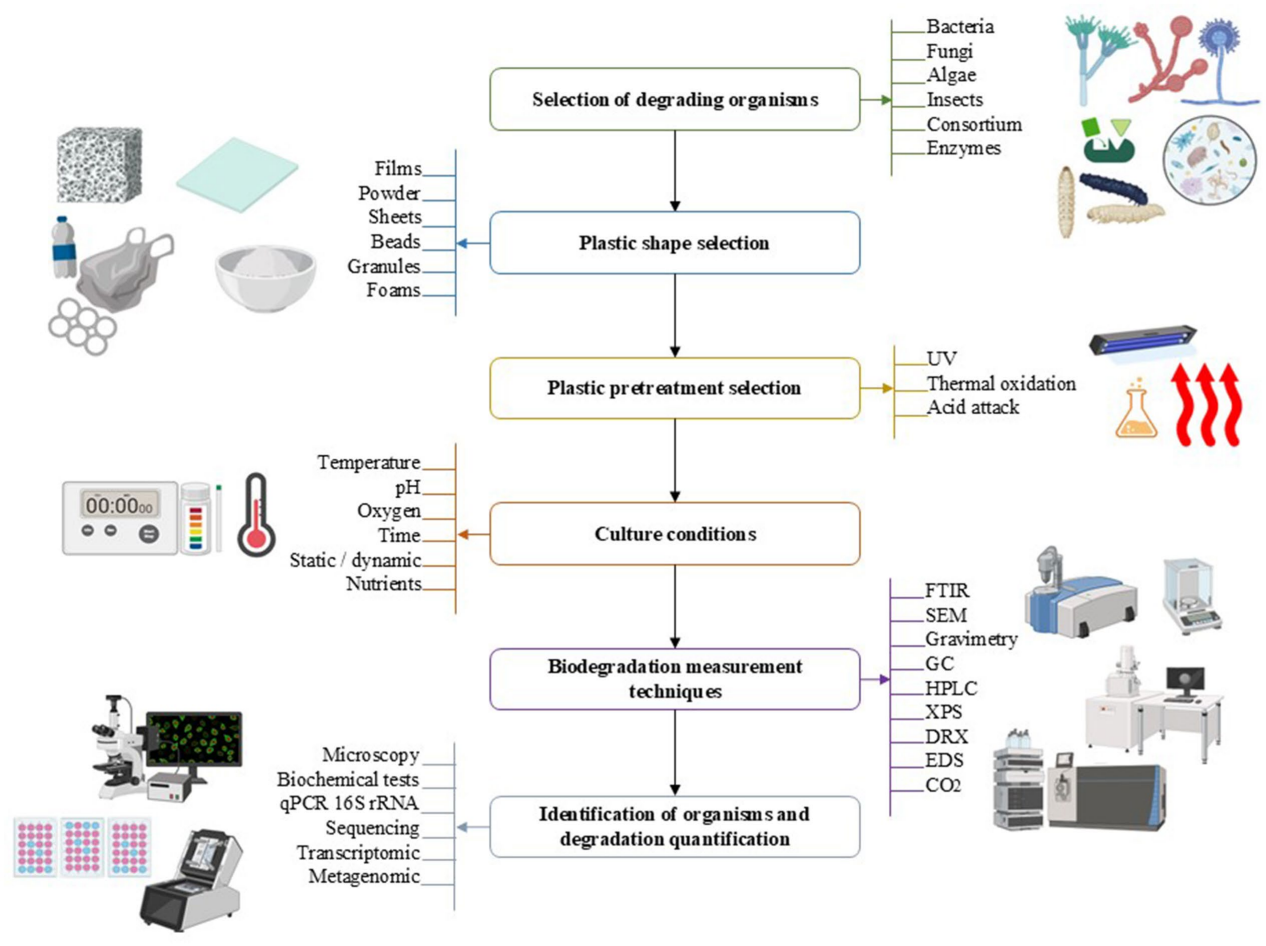
Suggested roadmap for the plastic biodegradation process.

To develop a consolidated methodology to maximize the efficiency of plastic biodegradation in agriculture, it is essential to consider several key factors: proper selection of degrading organisms, pretreatment of the plastic, and the use of accurate analytical techniques. A roadmap for the plastic biodegradation process is proposed in Figure 5. The selection of degrading organisms should be based on their ability to produce specific enzymes that can degrade the type of plastic in question. The combination of fungi and bacteria isolated from marine and terrestrial environments is effective in many studies (Fachrul et al., 2024; Wróbel et al., 2023; Mohy Eldin et al., 2022; Chigwada et al., 2023; Maheswaran et al., 2023). The use of microbial consortia, which include multiple species with complementary capabilities, can significantly improve biodegradation efficiency, or a specific strain or organism that needs to be tested as a plastic biodegrader must also be chosen. Pretreatment of plastic, such as UV exposure, thermal oxidation, and chemical treatment, can significantly increase its susceptibility to biodegradation. These treatments should be optimized for different types of plastics and experimental conditions to maximize their effectiveness. Further research should evaluate the effectiveness of these pretreatments in combination with microbial biodegraders to develop integrated and efficient processes.

Culture conditions must be controlled to optimize microbial activity and the production of degradative enzymes. Factors such as temperature, pH, nutrient availability, and oxygen concentration must be adjusted to maximize biodegradation efficiency. Continued research on optimizing these conditions for different microorganisms and types of plastics is crucial. The use of advanced analytical techniques is essential to assess the efficiency and extent of biodegradation. Techniques such as GC quadrupole mass spectrometry, GC flame ionization detection, FTIR, and SEM should be used to analyze changes in the chemical structure and morphology of the plastic during biodegradation. Despite significant advances in LDPE biodegradation, several areas require further research to improve the efficiency and applicability of these technologies. It is crucial to conduct comprehensive studies on the environmental impact and economic feasibility of LDPE biodegradation processes on an industrial scale. This includes assessing the toxicity of degradation products and the costs associated with the implementation of biodegradation technologies in different industrial contexts.

## 8 Perspectives in plastics biodegradation

The future of plastic biodegradation depends on the advancement of biotechnology, genetic engineering, and the implementation of sustainable solutions on a large scale. It is not enough to optimize degradation processes; it is essential to design, develop, and apply more efficient and cost-effective strategies that are applicable in various sectors such as agriculture, industry, and urban waste management. The application of genetically improved microorganisms may be an option for the development of more efficient, specific, and stable enzymes for the degradation of polymers such as polyethylene. The immobilization of enzymes on solid supports offers opportunities for their reuse and for continuous biodegradation processes. Biodegradation could be integrated with physical–chemical recycling procedures, within a holistic circular economy approach. Biodegradation derivatives, such as carbon dioxide, biomass, and/or recovered monomers, could be reused as inputs in production chains, thus ending the plastics cycle. For plastic biodegradation to be implemented, obstacles related to production costs and government regulations must be overcome.

## 9 Final reflections

From this review, it is established that it is essential to consider several measurements to assess biodegradation, covering different aspects: in the physical area, it is necessary to control the mass by measuring the percentage of weight lost from the plastic, make direct observations using SEM to monitor physical changes, and evaluate the level of crystallinity using TGA; in the chemical aspect, it is essential to use chromatography techniques such as HPLC and GC–MS to identify and quantify new compounds generated during biodegradation; and in the biological area, metabolic tests and identification of metabolic pathways should be carried out, applying omics sciences to study the genes and enzymes involved, as well as the interactions that are crucial, especially in the case of larvae whose microbiome plays a key role in biodegradation, since it was observed that there was a decrease in degradation capacity when antibiotics were applied to the larvae before subjecting them to the plastic biodegradation test. On the other hand, it is also important to evaluate the long-term sustainability of the consortia when trials with more than one microorganism are proposed. This integrated approach will allow a deeper and more accurate understanding of the biodegradation process, encompassing both the physical and chemical changes as well as the biological and microbial aspects involved.

According to the analysis of the methodologies currently used the most used organisms in recent years have been listed. Despite the progress made in the biodegradation of LDPE and other plastics used in everyday life including agriculture, fundamental aspects remain to be explored. Current research has identified promising organisms and methods, but it is clear that more in-depth and diversified studies are required to fully understand microbial interactions and their effectiveness under different environmental conditions. In addition, the disparity in the geographic distribution of research suggests an urgent need for more inclusive approaches that consider the global applicability of these technologies, especially in regions with fragile ecosystems. Furthermore, studies on microplastics and nanoplastics, i.e., on material undergoing biodegradation, are essential, since their effects can still be harmful and further treatments must be found for their complete mineralization. Therefore, this work lays a solid foundation but also leaves open several questions that future research will need to address to develop more effective biodegradable solutions adapted to various contexts. The remaining challenges include the need to deepen the understanding of the complex interactions between different microbial consortia, as well as their relationships with the environment, the enzymes involved, and their long-term efficacy under varying environmental conditions.

Detail of abbreviations in Supplementary Table S3.
