# Supplementary material for: Global perspectives on the biodegradation of LDPE in agricultural systems
Source: Front Microbiol. 2025 Jan 7;15:1510817. doi: 10.3389/fmicb.2024.1510817 (PMC11748793; doi:10.3389/fmicb.2024.1510817)
Supplement: Supplementary file 2 [file Table_2.docx]

**Table S1**. PE forms analyzed in the different studies

| PE Shape | Authors |
| --- | --- |
| Powder | (Vaksmaa et al., 2023)  (Tiwari et al., 2023)  (Pathak & Navneet, 2023)  (Skariyachan et al., 2021)  (Zampolli et al., 2021)  (Kim et al., 2023)  (Kong et al., 2024)  (Bernat et al., 2023)  (Ji et al., 2024)  (S. S. Yang et al., 2022)  (Adithama et al., 2023)  (Zampolli et al., 2021)  (Montazer et al., 2021)  (Tahani Gatea Obaid & Ihsan Flayyih Hasan AL-Jawhari, 2023)  (Ferrero et al., 2022)  (Zampolli et al., 2023) |
| Beads | (Adithama et al., 2023)  (Dey et al., 2020) |
| Granules | (Skariyachan et al., 2021)  (Wróbel et al., 2023)  (Maleki Rad et al., 2022)  (Kunlere et al., 2019) |
| Films | (H. Wang et al., 2023)  (Skariyachan et al., 2021)  (Ma et al., 2023)  (Khandare et al., 2022)  (Ji et al., 2023)  (Zhang et al., 2022)  (Huang et al., 2022)  (H. Zhang et al., 2023)  (Kong et al., 2024)  (Jeon et al., 2021)  (Jebashalomi et al., 2024)  (T. Zhang et al., 2024)  (Yuan et al., 2023)  (Zadjelovic et al., 2022)  (Wu et al., 2023)  (H. Lou et al., 2022)  (Rong et al., 2024)  (Hou et al., 2022)  (Adithama et al., 2023)  (Sanluis-Verdes et al., 2022)  (Puglisi et al., 2019)  (Scontri et al., 2022)  (Sáenz et al., 2019)  (Khandare et al., 2021)  (Kumar Gupta & Devi, 2019)  Khruengsai et al., 2021)  (Gupta & Devi, 2020)  (Maroof et al., 2021)  (Yao et al., 2022)  (Kundungal et al., 2021)  (Chaudhary et al., 2021)  (Soleimani et al., 2021)  Fibriarti et al., 2021)  (Gong et al., 2023)  (Samanta et al., 2020)  (Chaudhary et al., 2023)  (Khan et al., 2023)  (Sathiyabama et al., 2024)  Harrat et al., 2022)  (X. Zhang et al., 2023) |
| Sheets | (Zaman et al., 2024)  (Ji et al., 2023)  (XLantican et al., 2023)  (Yuan et al., 2023)  (Maheswaran et al., 2023)  (P. Wang et al., 2022)  (Hou et al., 2022)  (Sanniyasi et al., 2021)  (Mishra et al., 2024)  (El-Sayed et al., 2021)  (Perera et al., 2023)  (Joshi et al., 2022)  (Pinto et al., 2022)  (M. S. Khan et al., 2021) |
| Foams | (Peng et al., 2022)  (Lou et al., 2021)  (L. Yang et al., 2021)  (Gerritse et al., 2020)  (J. Wang et al., 2022) |
| Bags | (Jayan et al., 2023)  (Mohy Eldin et al., 2022)  (Gerritse et al., 2020)  (Nnaji et al., 2021)  (Nademo et al., 2023)  (Poma et al., 2022) |
