# Supplementary figures and images for: Global perspectives on the biodegradation of LDPE in agricultural systems
Source: Front Microbiol. 2025 Jan 7;15:1510817. doi: 10.3389/fmicb.2024.1510817 (PMC11748793; doi:10.3389/fmicb.2024.1510817)

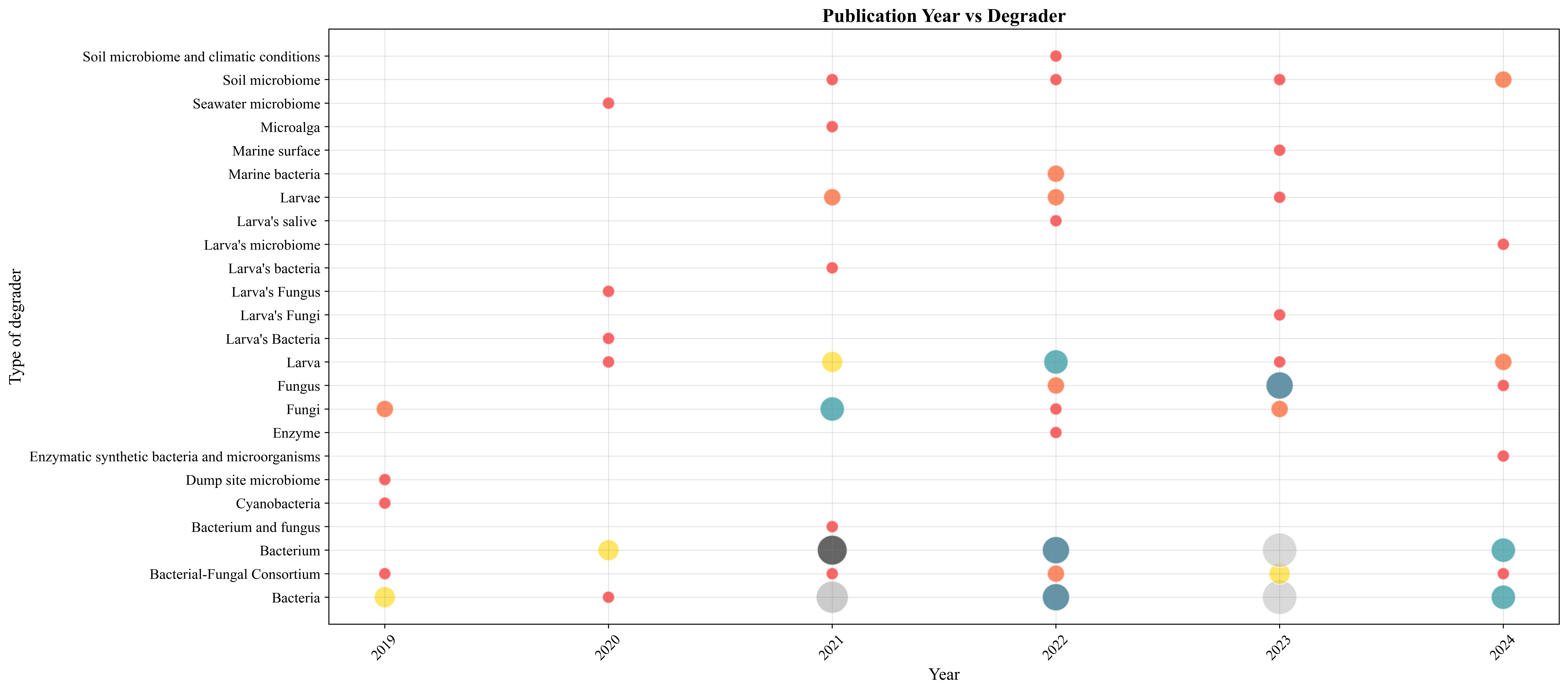

Supplement: Supplementary file 4 [file Image_1.jpeg]

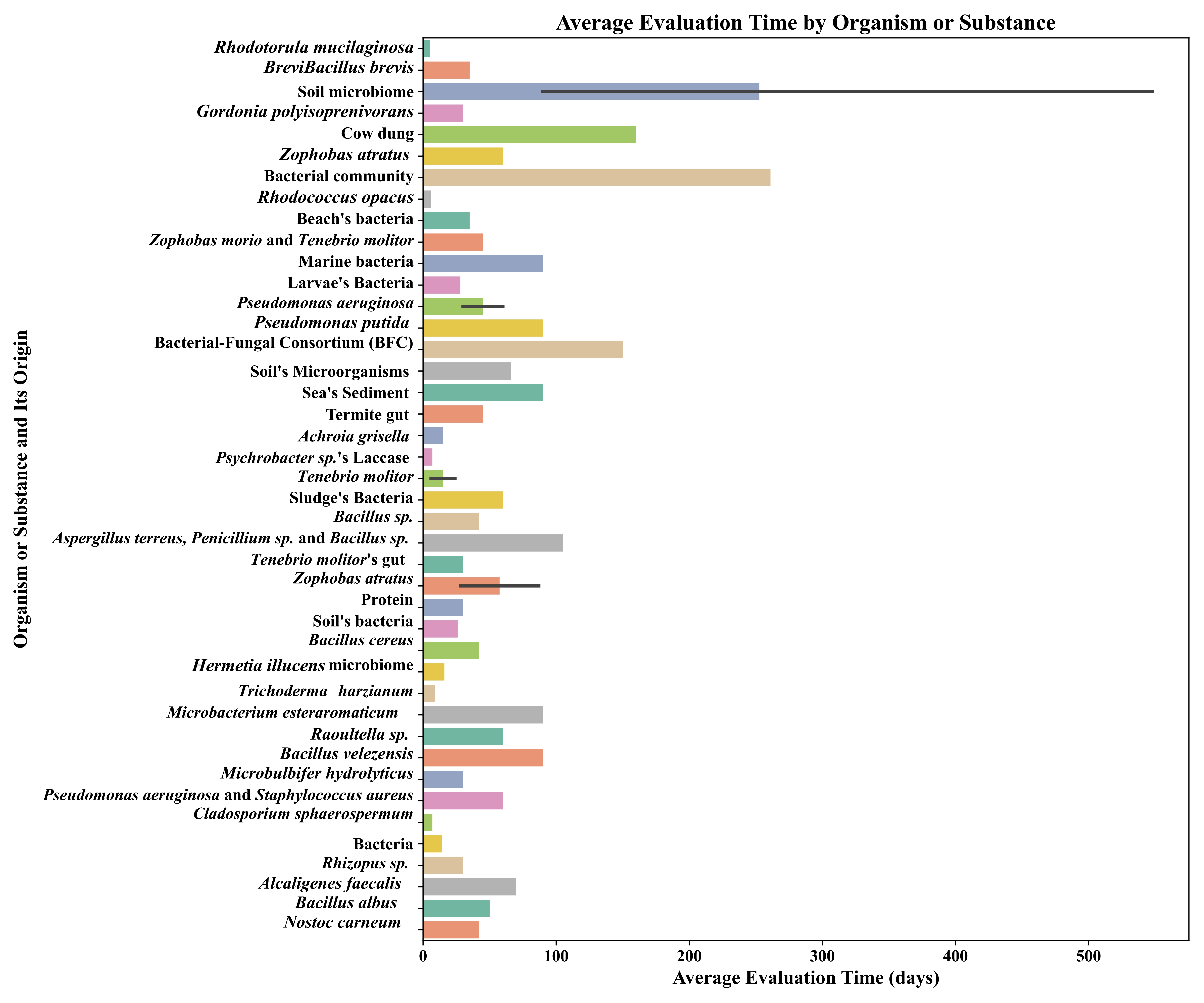

Supplement: Supplementary file 5 [file Image_2.jpeg]

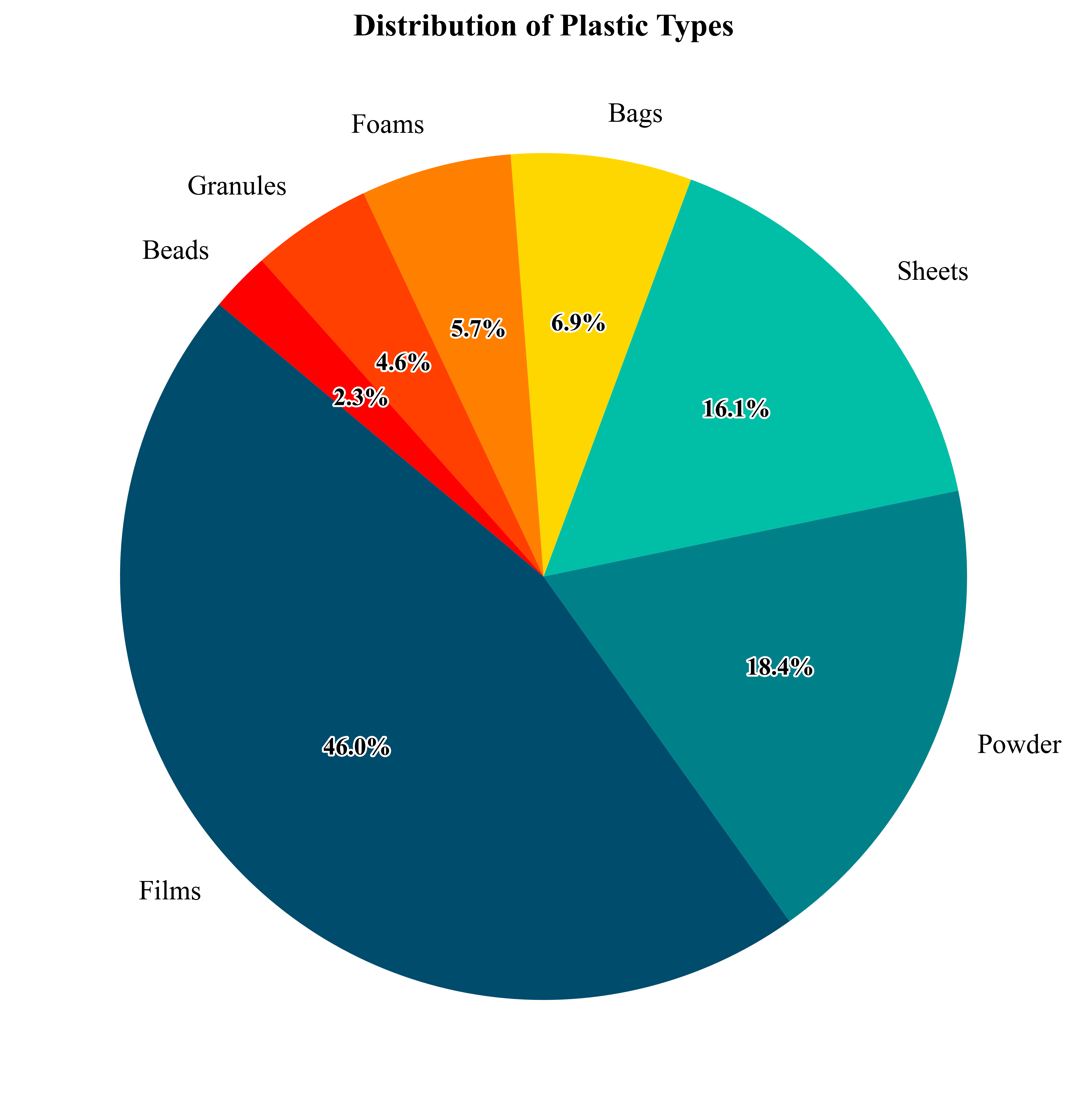

Supplement: Supplementary file 6 [file Image_3.png]
